# Immunosuppression during Acute Infection with Foot-and-Mouth Disease Virus in Swine Is Mediated by IL-10

**DOI:** 10.1371/journal.pone.0005659

**Published:** 2009-05-21

**Authors:** Fayna Díaz-San Segundo, Teresa Rodríguez-Calvo, Ana de Avila, Noemí Sevilla

**Affiliations:** 1 Centro de Investigación en Sanidad Animal, INIA,Valdeolmos, Madrid, Spain; 2 Centro de Biología Molecular Severo Ochoa, Cantoblanco, Madrid, Spain; University of Toronto, Canada

## Abstract

Foot-and-mouth disease virus (FMDV) is one of the most contagious animal viruses, causing a devastating disease in cloven-hoofed animals with enormous economic consequences. Identification of the different parameters involved in the immune response elicited against FMDV remains unclear, and it is fundamental the understanding of such parameters before effective control measures can be put in place. In the present study, we show that interleukin-10 (IL-10) production by dendritic cells (DCs) is drastically increased during acute infection with FMDV in swine. *In vitro* blockade of IL-10 with a neutralizing antibody against porcine IL-10 restores T cell activation by DCs. Additionally, we describe that FMDV infects DC precursors and interferes with DC maturation and antigen presentation capacity. Thus, we propose a new mechanism of virus immunity in which a non-persistent virus, FMDV, induces immunosuppression by an increment in the production of IL-10, which in turn, reduces T cell function. This reduction of T cell activity may result in a more potent induction of neutralizing antibody responses, clearing the viral infection.

## Introduction

Viruses use a variety of strategies to avoid recognition by the host immune system [1], [2], [3]. The active induction of immune suppression is one mechanism by which viruses escape clearance [4]. Several viruses are known to target dendritic cells (DCs) and impair antiviral T cell responses [5], [6], [7], [8], [9], [10]. DCs are the most potent of the professional antigen-presenting cells (APCs), with the unique capacity to prime naive T cell reponses and to maintain immunity acting as sentinels of the immune system [11], [12]. It has been demonstrated that DCs can directly indentify and recognize pathogens via Toll-like receptors (TLRs), resulting in the subsequent up-regulation of expression of co-stimulatory molecules, proinflammatory cytokines, chemokines and enhancement of antigen presentation [13], [14]. These processes allow these cells to mature, and be capable of eliciting adaptive immunity [15]. The status of DCs, mature or immature, affects their ability to uptake, process, and migrate to the draining lymph nodes, where they present the antigen to effector T cells, ultimately mounting an effective cell-mediated response [16]. It has been recently reported that interleukin 10 (IL-10) has an important role in the induction of immunosuppression *in vivo* [17], [18]. IL-10 inhibits a broad spectrum of cellular responses. Among them, it suppresses the function of APCs and T cells by inhibiting co-stimulation, MHC class II expression, and chemokine secretion [19], [20]. IL-10 also has anti-inflammatory activity and stimulates cellular proliferation of B cells [19], [21].

Foot-and-mouth disease (FMD) is a highly contagious disease with high morbidity in cloven-hoofed animals, including important livestock species such as cattle and swine. FMD is caused by FMD virus (FMDV), the only member of the *Picornaviridae* family, genus *Aphtovirus* [22]. The virus genome consists of a positive stranded RNA molecule of about 8,500 nucleotides, which encodes four structural proteins (VP1, VP2, VP3 and VP4) and nine non-structural proteins (L/L′, 2A, 2B, 2C, 3A, 3B, 3C and 3D) [23]. FMDV infection in susceptible animals results in viremia 1–3 days post-infection, followed by a rapid onset of clinical disease, with an incubation period of 2–10 days. Protection against FMDV is often associated with the induction of high levels of circulating neutralizing antibodies in serum, and these neutralizing antibodies can be found as early as 4 days post-infection, although this response does not ensure clinical protection, and animals with low levels of neutralizing antibodies may nevertheless be protected [24]. This indicates that cell-mediated immunity may play a role in the elimination of the virus. Thus, given that exposure to the virus results in the production of T-cell dependent neutralizing IgG class antibodies, and subsequent T-cell dependent memory, it is clear that CD4^+^ cells must be stimulated [25], [26], [27], [28].

During acute infection with FMDV in swine, we and others have reported an immunosuppressive stage, characterized by T cell unresponsiveness [29], [30]. Throughout this stage, a severe but transient lymphopenia affecting all T cell subsets correlates with the appearance of viremia. However, when viremia is cleared, both, the number of lymphocytes and the altered T cell function, start to recuperate normal levels. These effects on the early host immune response provide the perfect conditions for viral spread through the organism, and subsequent shedding to the environment. The mechanism by which the virus induces immunosupression is not completely understood. We have described infection of lymphocytes by FMDV serotype C *in vivo* [29] as a possible cause of T cell functional impairment. However, the implication of other cells and/or other mechanisms of the immune system deserve to be deeply analyzed. It is of great interest to study the interaction of FMDV with APCs, and more precisely, DCs. The interaction of FMDV with DCs has been previously described by others who have reported an absence of detectable FMDV replication in mature DCs *in vitro* [30], [31], [32]. Nevertheless, it is important to determine the interaction of FMDV with DC progenitors, and how these cells function during acute infection with FMDV. In this report, we trace the events following infection of DCs and its progenitors by FMDV serotype C. First, we show that FMDV infects DCs progenitors CD172^+^ cells, but not differentiated DCs *in vitro*. This infection leads to inhibition of maturation and antigen presentation of DCs. Second, we show that antigen presentation of DCs isolated *ex vivo* from FMDV infected swine is impaired. Finally, we reveal that production of IL-10 by DCs plays a critical role in impairing activation of T cells during acute infection with FMDV in swine.

## Results

### FMDV infects immature MoDCs

**Figure 1:**
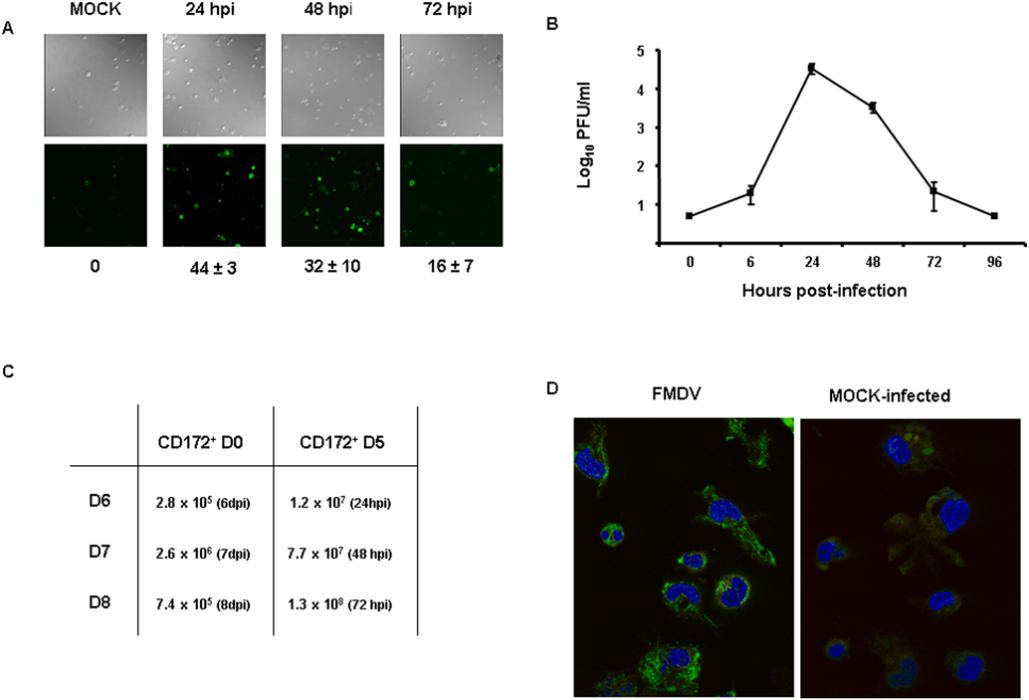
Replication of FMDV C-S8c1 in CD172^+^- MoDC. CD172^+^ cells were purified from swine PBMCs (see Material and Methods) and put in culture with rpGM-CSF and rpIL-4. The cells were infected either at D0 (just after purification) or D5 (5 days in culture with rpGM-CSF and rpIL-14) with FMDV C-S8c1 at moi of 10 PFU/cell. A. Staining of CD172^+^ cells infected at D0 with a monoclonal antibody against FMDV 3D (3H11) (green). At 24, 48 and 72 p.i. cells were fixed and analyzed by immunofluorescence microscopy. The percentage of positive cells for FMDV 3D in the field (average of at least 10 fields±SD) is indicated in the lower part of each panel. B. FMDV growth curve in CD172^+^ cells infected at D0. The results are representative of three independent experiments. C. Viral RNA expressed as the number of FMDV RNA molecules quantified by real time RT-PCR (described in Material and Methods) per 10^4^ cells. In the left column is indicated the day post-differentiation at which the viral RNA was extracted from FMDV-infected MoDCs. CD172^+^ D0 indicates CD172^+^ MoDCs infected with FMDV C-S8c1 at D0 post-differentiation; CD172^+^ D5 indicates CD172^+^ MoDCs infected at D5 post-differentiation. In the right column is indicated in braches the corresponding time post-infection. D. Confocal microscopy was used to analyze the expression of FMDV 3D protein (green) at 6 hpi of CD172^+^ cells infected with FMDV at D5 (5 days post-differentiation). Nuclei were stained with DAPI (blue). The micrographs are representative of at least three independent experiments.

To gain a more comprehensive understanding of the mechanism(s) by which FMDV infected swine are immunosuppressed during acute infection [29], [30], we studied the susceptibility of porcine monocyte-derived DCs (MoDCs) to FMDV infection *in vitro*. CD172^+^ cells were generated from peripheral blood mononuclear cells (PBMCs) and were cultured in the presence of rpGM-CSF and rpIL-4. In the first set of experiments, CD172^+^ cells were infected with FMDV C-S8c1 at MOI of 10 at day 0 (D0) of development, washed with phosphate buffer pH 6.0 to eliminate any virus bound to cell membranes that may account for infectivity, incubate, in the presence of rpGM-CSF and rpIL-4 and, at different time-points, the supernatants of the cells were harvested and the cells were fixed. By 24 hours post infection (hpi) a high percentage of cells (44±3) were seen to express FMDV non-structural protein 3D by immunoflourescence (Fig. 1A). The presence of positive cells was maintained by 48 hpi (32±10) and by 72 hpi the number of cells expressing FMDV 3D started to decline (16±7) (Fig. 1A), suggesting a productive infection of MoDCs. This result was confirmed by the observation of a 4–5 log_10_-fold increase in the virus titer determined by plaque assay in susceptible BHK-21 cells at 24 to 48 hpi, but clearance by 72 hpi (Fig. 1B). Neither positive cells for FMDV 3D nor virus were detected by 96 hpi. Nevertheless, we use real time RT PCR at later times post-infection to determine if by a more sensitive technique we could detect any viral RNA. The amount of FMDV RNA detected at day 6, 7 and 8 post-infection did not increase, indicating that active viral replication might be hampered (Fig. 1C) when DCs were infected at D0. Thus, these results indicate that FMDV is able to infect CD172^+^ cells but viral replication may be aborted along with cell differentiation.

To clarify whether FMDV could infect at multiple stages along a development pathway of MoDCs, we repeated the same earlier experimental designed but using CD172^+^ cells previously cultured with rpGM-CSF and rpIL-4 during 5 days (D5). Viral production by plaque assay on BHK-21 cells was not detected even at the earliest time post-infection tested (6 and 24 hpi) (data not shown). However, by confocal microscopy cells were expressing non-structural viral proteins (Fig. 1D), and by real time RT PCR viral RNA was detected at 24, 48 and 72 hpi, showing an increased in the amount of viral RNA (Fig. 1C). This suggests that the virus was able to replicate but there was not viral particles produced that could be detected by plaque assay. To determine if virus was produced even though no virus was detected by plaque assay on BKH-21 cells, supernatants of D5 CD172^+^ cells infected with FMDV were harvested at different time points (6, 24 and 48 hpi) and were subjected to two blind passages in BHK-21 cells. Cytopathic effect was detected after two passages in BHK-21 of supernatants only from cultures at 6 hpi, but at no at other times post-infection, indicating that CD172^+^ cells at later time post-differentiation (D5 post-culture) are not productively infected by FMDV, therefore an abortive infection is taking place.

### FMDV interferes with MoDC development *in vitro*

**Figure 2:**
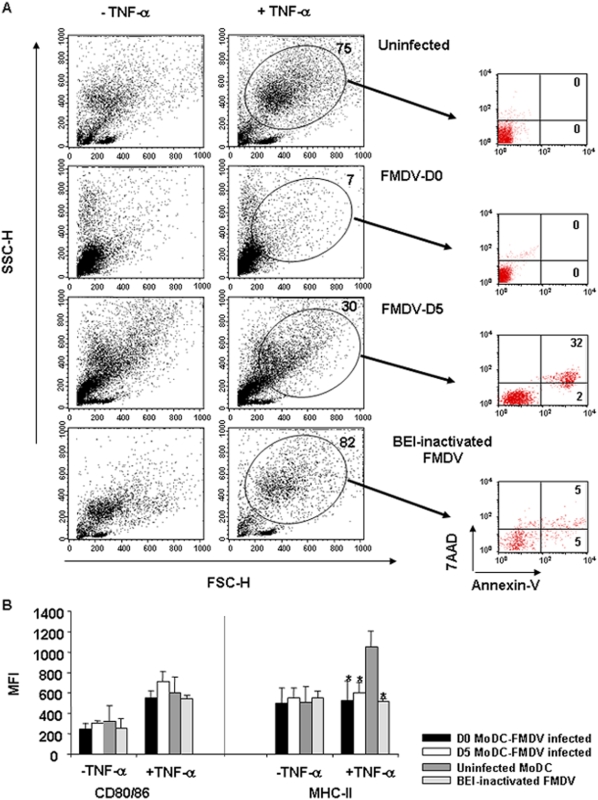
FMDV interferes with MoDC development *in vitro*. A. Dot plots show forward scatter (FSC) and side scatter (SSC) for uninfected, FMDV-infected at D0 and FMDV-infected at D5 before TNF-α stimulation (−TNF-α) and upon TNF-α treatment (+TNF-α). Note the lack of size increased in FMDV-infected MoDC after TNF-α at either D0 or D5 compared with uninfected control cells. It is shown the population with MoDCs phenotype in a circle and the percentage of this population is indicated. In the right panel, the viability and apoptosis of MoDCs analyzed by annexin-V and 7AAD staining is shown. This is the results from a representative experiment (n = 4) B. The mean fluorescence intensity (MFI) of surface molecules expressed on MoDCs. Each bar represents the MFI of a given surface molecule (CD80/86 or MHC class II) before and after TNF-α addition. Black bars: FMDV-infected MoDC at D0; white bars: FMDV-infected MoDC at D5; gray bars: uninfected cells. Data are average of four independent experiments±SD. Asterisks denote a statistically significant reduction in FMDV-infected MoDC compared with uninfected controls (student *t* test, p<0.05).

In order to determine if the viral infection had any effect on cell differentiation or in the expression of cell surface proteins important in T-cell stimulation, we next analyzed the effect(s) of viral infection on cell differentiation and expression of co-stimulatory molecules (CD80/86) and MHC class II. CD172^+^ cells infected with FMDV at D0 or D5 were cultured in the presence of rpGM-CSF and rpIL-4 until day 7 (immature phenotype), and then TNF-α was added to the culture for an additional 24 h to induce complete maturation. We initially studied by flow cytometry the profile of forward scatter (FSC) and side scatter (SSC) in order to determine cell size and granularity evolution in the process of maturation. Uninfected cultures underwent a dramatic increase in size and granularity after TNF-α treatment, according to mature MoDC phenotype (Fig. 2A). In stark contrast, when cells were previously infected with FMDV, either at D0 or D5, the FACS analyses did not show an increase in size and granularity after adding TNF-α (Fig. 2A). Interestingly, CD172^+^ cells matured in the presence of rpGM-CSF and rpIL-4 according to a normal pattern until FMDV was added to the culture (compare Fig. 2A, panel uninfected and FMDV-D5 without TNF-α). Due to the fact that FMDV is a highly cytopathic virus in several lines, we evaluated apoptosis and viability of mature MoDCs (after TNF-α treatment) by annexin-V staining and 7AAD exclusion. Infection of MoDCs by FMDV did not decrease cell viability after complete maturation compared with uninfected or BEI-inactivated FMDV MoDCs (Fig. 2A). We therefore analyzed the expression of co-stimulatory molecules and MHC class II on FMDV infected at different stages of maturation (D0 and D5), and uninfected CD172^+^ cells. After incubating with TNF-α, the mean fluorescence intensity of CD80/86 was increased in FMDV infected cells to similar levels than uninfected cells (Fig. 2B). However, MHC class II expression did not increase on FMDV infected cells upon TNF-α treatment (Fig. 2B), making this difference statistically significant (p<0.05) as compared with the uninfected MoDCs after adding TNF-α. Together, the data show that FMDV may disrupt MoDCs at multiple stages along a development pathway, even when they showed only abortive infection (D5 infected MoDCs). To determine if viral replication is required to interfere with MoDCs development, BEI-inactivated FMDV was added to MoDCs at D0 and D5 post-differentiation. These cultures underwent an increase in size and granularity as well as up-regulation of CD80/86 and MHC-II similar to uninfected controls (Fi. 2A, B), indicating that virus replication is needed to trigger impair MoDCs development.

### FMDV infection impairs MoDC function

**Figure 3:**
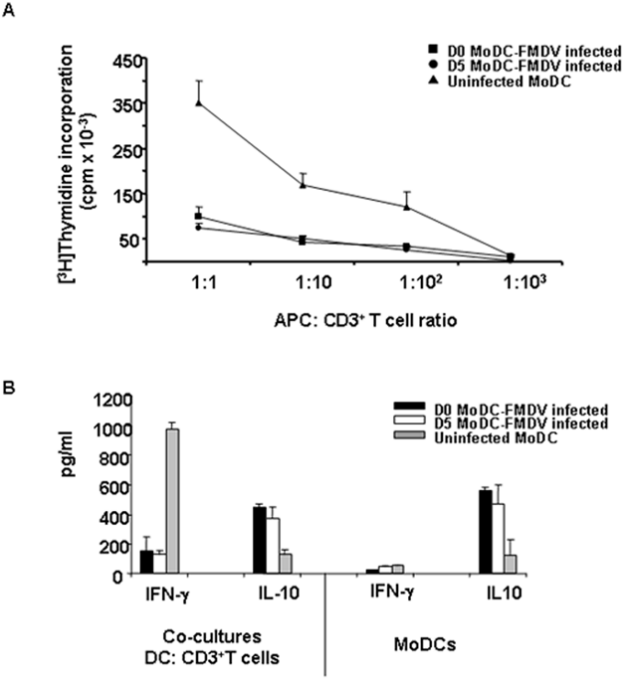
FMDV infection impairs T cell function. A. The allostimulatory capacity of uninfected MoDCs (triangles), FMDV C-S8c1-infected MoDCs at D0 (squares) and FMDV C-S8c1-infected MoDCs at D5 (circles) is indicated at different CD3^+^ T cells: MoDC ratios. Uninfected or infected MoDCs were irradiated and used as stimulators cells for allogeneic CD3^+^ T cells. Proliferation was measured in cpm (average cpm±SD) after [^3^H]Thymidine incorporation and are representative of three independent experiments. B. 72 hours after the onset of the co-cultures MoDCs and CD3^+^ T cells (ratio 1∶10), or MoDCs, supernatants were collected and analyzed for IFN-γ and IL-10 production by quantitative ELISA. The figure shows mean concentration values (pg/ml). Black bars: FMDV-infected MoDC at D0; white bars: FMDV-infected MoDCs at D5; gray bars: uninfected controls.

The previous results indicated that the process of maturation of the MoDCs upon FMDV exposure was impaired. However, these results alone do not allow for definitive conclusions on the maturation status of DCs in terms of T-cell stimulatory activities [33]. To test whether the capacity of FMDV to reduce the surface expression of MHC class II paralleled an inability to stimulate the proliferation of T cells, we investigated the ability of MoDCs infected with FMDV D0 and D5 to induce the proliferation of T cells in a primary allogeneic mixed lymphocyte reaction (MLR). MoDCs previously infected with FMDV at D0, D5 or uninfected, were maturated by adding TNF-*α* and incubated with allogeneic CD3^+^ T cells for 3 days. As shown in figure 3A, DCs that have been exposed to FMDV were not efficient at stimulating a primary MLR, whereas DCs from uninfected controls were very effective. These results demonstrate that infection with FMDV resulted in a functional deficit in APCs that aborts a primary MLR. To further examine the mechanisms involved in the functional deficit of DCs that have been infected by FMDV, we looked for the production of IFN-γ, as an antiviral cytokine, and IL-10 as an immunosuppressive cytokine, in the co-cultures of MoDCs infected at D0, D5 or uninfected, and T cells. Co-cultures of uninfected MoDCs with T cells (1:10 APC: CD3^+^ T cells ratio) produced high amounts of IFN-γ whereas co-cultures of FMDV infected MoDCs with T cells did not produce significant amount of IFN-γ (uninfected MoDCs: 975±100 pg/ml; FMDV D0 infected MoDCs: 150±30 pg/ml; FMDV D5 infected MoDCs: 125±45 pg/ml) (Fig. 3B). By contrast, the amount of IL-10 produced in the co-cultures of FMDV infected MoDCs either at D0 or D5 with T cells were 4-fold higher than uninfected MoDCs co-cultures with T cells (uninfected MoDCs: 125±20 pg/ml; FMDV D0 infected MoDCs: 450±75; FMDV D5 infected MoDCs: 375±34 pg/ml) (Fig. 3B). To determine whether IL-10 could be produced by MoDCs alone, or upon interaction with T cells, supernatants of MoDCs cultures infected at two different stages of maturation, D0, D5 or uninfected, were tested for the presence of IL-10 and IFN-γ production. Interestingly, uninfected MoDCs did not produce either IFN-γ or IL-10 whereas FMDV infected MoDCs (at D0 or D5) produced substantial amount of IL-10 but no IFN-γ (Fig. 3B). These results indicate that FMDV induces production of IL-10 in DCs, and more likely, IL-10 inhibits activation of T cells by DCs.

### DCs *ex vivo* from FMDV infected pigs do not activate T cells

**Figure 4:**
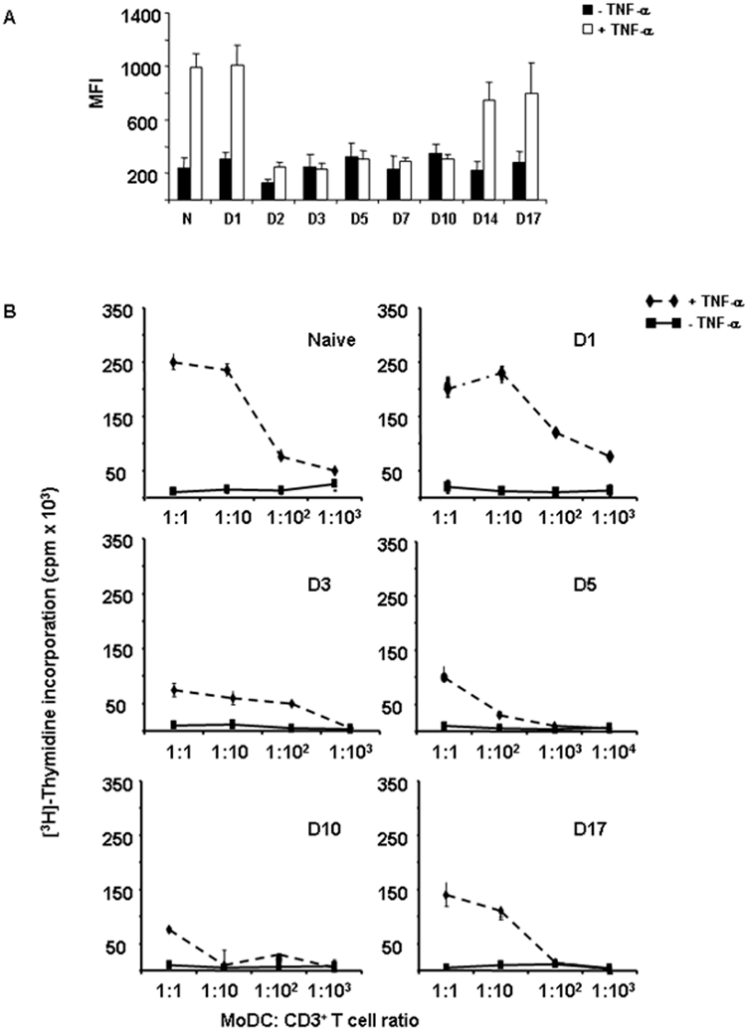
MoDCs from FMDV-infected swine do not up-regulate CD80/86 and do not stimulate T cells. A. Flow cytometric analyses were performed to measure the expression of CD80/86 on MoDCs differentiated from PBMCs isolated from FMDV C-S8c1-infected swine. It is indicated the mean fluorescence intensity (MFI) of CD80/86 on MoDCs from naïve animals (N) and MoDCs from FMDV C-S8c1-infected pigs at different times post-inoculation (indicated as D1, D3, D5, D10 and D17) before treatment with TNF-α (black bars) and after treatment with TNF-α (white bars). B. The allostimulatory capacity of MoDCs isolated from FMDV-infected swine either treated with TNF-α (diamonds and dashed line) or untreated (squares) at different times post-infection (indicated in each graph). MoDCs were irradiated and used as stimulator cells for allogeneic CD3^+^ T cells at the T cell/DC ratio indicated. Proliferation was measured in cpm (average cpm±SD) after [^3^H]Thymidine incorporation. Data is representative of three independent experiments.

Based on our initial *in vitro* findings that FMDV-infected MoDCs do not stimulate T cells, we hypothesized that the loss of T cell function observed during acute FMDV infection in pigs would be mediated by MoDCs. We therefore examined MoDCs obtained *ex vivo* from FMDV infected pigs. Twenty-four Large White×Landrace pigs were inoculated with 10^5^ PFU of FMDV C-S8c1 and four pigs were used as uninfected controls (see Materials and Methods). All the inoculated animals developed clinical signs including fever and vesicles in tongue, feet and snout and severe viremia, with the peak between day 2 and 3 post-infection that was cleared by day 10 pi (data not shown). PBMCs were purified at different days (1, 3, 5, 7, 10 and 14 pi) and CD172^+^ cells were sorted to be cultured with rpGM-CSF and rpIL-4. At day 7 of culture, TNF-α was added and kept for additional 24 hours. First, we determined the immunophenotype of MoDCs by quantifying the expression of co-stimulatory molecules and MHC class II. We found that, in contrast to the *in vitro* data, MoDCs obtained *ex vivo* from infected swine do not up-regulate CD80/86 after TNF-α treatment (Fig. 4A). At day 1 pi, MoDCs still responded to the stimuli but at day 2 pi the MoDCs were not able to up-regulate co-stimulatory molecules. Although the viremia was cleared by day 10 pi the MoDCs started to recover by day 14 pi. Interestingly, MHC class II was up-regulated on *ex vivo* MoDCs from FMDV infected swine to values reached by naïve MoDCs (data not shown). Based on these results, we assessed the functionality of MoDCs in primary MLR. We discovered that *ex vivo* MoDCs from FMDV infected pigs at day 3 pi were not able to stimulate T cells, and this lack of MoDC function lasted until day 17 pi (the latest time observed in this report) (Fig. 4B). This data suggests that the immunosuppression described in FMDV C-S8c1 infected swine [29] may be mediated partially by the lack of MoDC function.

A main question to be addressed was if CD172^+^ cells isolated from infected animals, and used as a source for the generation of MoDCs, were infected by FMDV. To test this, we took supernatants of CD172^+^ cells at different times post-differentiation. We were not able to detect the virus replication, neither by plaque assay on BHK-21 cells nor in blind passages in BHK-21 cells, (data not shown). This suggests that FMDV does not infect productively MoDCs *in vivo*, at least for the methods used to detect viral infection production.

### IL-10 suppresses T cell proliferation

**Figure 5:**
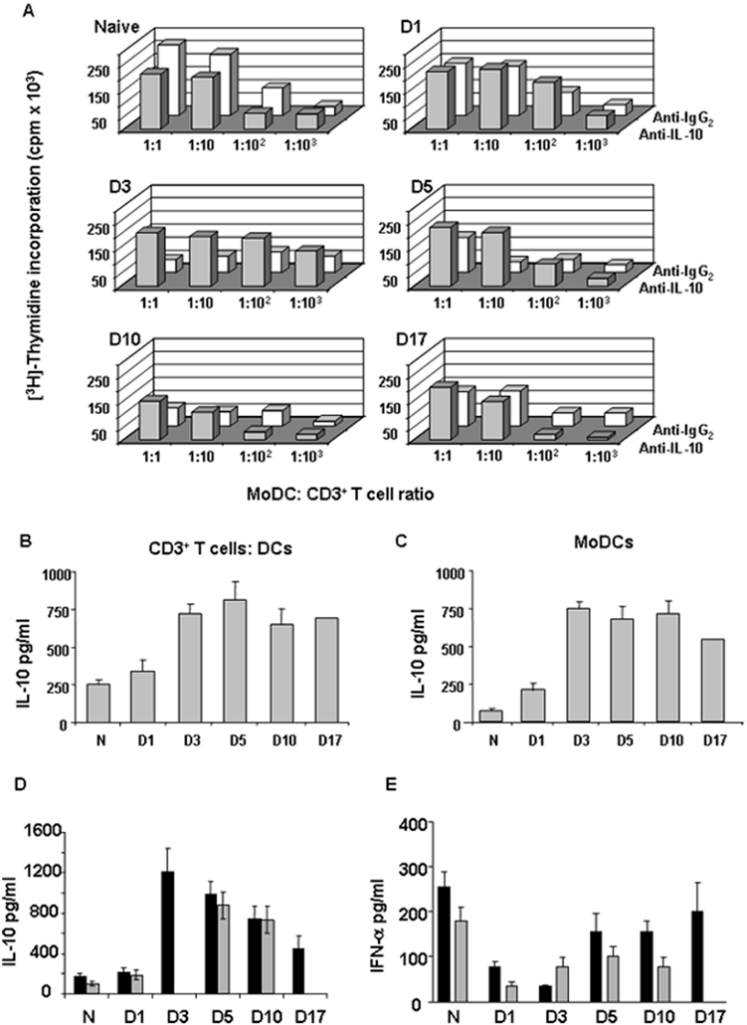
Anti-IL-10 restores T cell activation by MoDCs. A. The allostimulatory capacity of MoDCs from FMDV-infected swine (D1 to D17 pi) and naïve swine is evaluated in the presence of anti-IL-10 monoclonal Ab (gray squares) or an irrelevant antibody (white squares) (see material and Methods). MoDCs were irradiated and used as stimulator cells for allogeneic CD3^+^ T cells at the T cell/DC ratio indicated. Proliferation was measured in cpm after [^3^H]Thymidine incorporation. Data is representative of three independent experiments. B. IL-10 production by co-cultures of MoDC from FMDV-infected swine and T cells from a naïve pig. 72 h after the onset of the co-culture (MoDC: CD3^+^ T cell ratio of 1∶10), supernatants were collected and analyzed for IL-10 production by quantitative ELISA. The figure shows mean concentration values (pg/ml). C. IL-10 production by MoDCs from FMDV-infected swine. IL-10 production by cultures of MoDCs was determined by quantitative ELISA. It is expressed in pg/ml±SD. D. IL-10 produced in sera from FMDV-infected swine at different times post-inoculation. IL-10 was detected by ELISA. Each bar corresponds to one animal and it is expressed as pg/ml±SD. E. IFN-α produced in sera from FMDV-infected swine at days 1, 3, 5, 10 and 17 post-inoculation by ELISA. N, indicates naïve animals. Each bar corresponds to one animal and it is expressed as pg/ml±SD.

One possible factor inducing suppression of T cell proliferation by *ex vivo* MoDCs is IL-10, mainly because we have shown that this cytokine is highly produced by *in vitro* FMDV infected MoDCs, and due to the immunosuppressive role on T cell proliferation of this cytokine [34]. Therefore, the amount of IL-10 being produced in co-cultures of T cells and MoDCs isolated *ex vivo* from FMDV infected swine at different times post-infection was evaluated. We found that IL-10 was highly produced in co-cultures of MoDCs isolated *ex vivo* between days 3 and 17 pi and T cells of a naïve animal (average value of 720 pg/ml in FMDV infected pigs versus 250 pg/ml in naïve animals) (Fig. 5B), when the maximum inhibition of T cell proliferation was observed (see figure 4B). Thus, all the data pointed out to a role of IL-10 in T cell suppression. To confirm a possible role of IL-10, we next determined whether antibody blockade of IL-10 could restore T cell activation. T cells and MoDCs were cocultured in the presence of anti-porcine IL-10 monoclonal antibody for a period of 3 days. Interestingly, T cell activation was almost restored to the level shown by naïve animals in all the co-cultures that were previously inhibited (Fig. 5A). Surprisingly, although by day 10 and 17 we were able to observe a certain level of restoration of T cell stimulation, it never achieved the levels of the controls (Fig. 5A). Therefore, IL-10 seems to be causing T cell inhibition. One possibility may be that MoDCs were producing IL-10, as we have previously shown with *in vitro* FMDV infected MoDCs, or that they induce the secretion of this cytokine upon an interaction with T cells. To gain insight into the cell subtype responsible for IL-10 secretion, supernatants of MoDCs produced from CD172^+^ cells isolated from FMDV infected pigs and, the amount of IL-10 secreted was determined. Notably, MoDCs were producing a high amount of IL-10 (Fig. 5C), indicating that IL-10 is produced by MoDCs *in vivo*. Actually, the amount of IL-10 in serum of FMDV infected swine is significantly high compared with naïve animals (Fig. 5D). An important cytokine that has been implicated in mediating immunosuppression in acute viral infections is IFN-α. To determine any role of this cytokine in the immunosuppressive stage of FMDV-infected swine, we have looked at the level of IFN-α in serum of FMDV-infected pigs at different times post-inoculation by quantitative ELISA. The amount of IFN-α in infected swine is similar or even below the levels found in naïve animals (Fig. 5 E), indicating that IFN-a is not playing a relevant role in immunossuppresion during FMDV infection. All these data together suggest that IL-10 is contributing to the immunossuppression observed in FMDV infected swine during the peak of the viremia but not IFN-α.

## Discussion

In this study, we report for the first time that IL-10 signaling *in vivo* leads to immunosuppression in FMDV infected swine. Our previous data demonstrated that upon challenge of swine with FMDV C-S8c1, a state of immunosuppression associated with generalized lymphopenia is imposed rapidly after infection [29], being the animals recovered at later times post-infection. IL-10, a interleukin that inhibits a broad spectrum of cellular responses, has been reported to have a role in inducing immunosuppression *in vivo* [17], [18]. In this paper, we show that the early immunosuppression observed in FMDV infected pigs is associated with impaired MoDC function and high production of IL-10. The implication of IL-10 in the induction and maintenance of immunosuppression is demonstrated by the reversion of MoDC function upon blockade of IL10-IL-10R signaling. Furthermore, we show that exposure of MoDCs to FMDV *in vitro* impairs MoDC function and causes the production of high amounts of IL-10, although no viral replication is required. Indeed, after FMDV infection in pigs, viral replication in MoDCs was not found. Thus, it is possible that the generalized state of T cell unresponsiveness shown by FMDV-infected swine at early times post-infection is mediated by IL-10 produced by MoDCs. We speculate that IL-10 modulates MoDC function early after infection and, as a result, dictates the nature of the cytokine response induced early during antiviral immunity, favoring a Th2 cell/cytokine-like environment inducing FMDV specific neutralizing antibodies.

Our data indicates that FMDV C-S8c1 is able to infect productively MoDC-CD172^+^ progenitors cells *in vitro*, interfering with the development of mature MoDCs and mount an efficient immune response. In addition, CD172^+^ cells at later time post-maturation (immature MoDCs, D5) can be infected by FMDV but no viral production was detected and results in an abortive replication. In our hands, and according to previous reports [31], [32], [35], [36], mature MoDCs are not infected by FMDV (data not shown). The inhibitory effect that FMDV has on MoDC progenitor cells appears to be associated with its ability to infect progenitor or immature CD172^+^ cells. The way by which FMDV directly disrupt cell differentiation may be by different mechanisms. One mechanism could be by selectively inhibiting the transcription of specific genes. In this sense, the virus can abort the differentiation (luxury) function of a cell without affecting its vital “housekeeping” functions or inducing cell death [37], [38], [39]. Another mechanism by which FMDV may interfere with progenitor cells maturation is through the indirect induction of an inhibitory factor (or factors). In support of this second mechanism, induction of IFNα/β in mice by lymphocytic choriomeningitis virus (LCMV) infection has been reported to interfere with DC differentiation *in vivo* [40]. However, we have looked at IFN-α production in FMDV-infected MoDC cultures and the levels of IFN-α are very low or below the detection limit of the technique used (data not shown). Actually, this data agrees with previous report in which MoDCs exposed to FMDV do not produce IFN-α (Nfon….).

Although MoDC differentiation is accompanied by an increased expression of several surface molecules that facilitates T cell stimulation, the regulation of MHC class II molecule transport plays a central role in developmentally restricting antigen presentation [42], [43]. CD172^+^ cells at both early and late times *in vitro* infected by FMDV showed a marker reduction in the expression of MHC class II molecules (Fig. 2B). Importantly, MoDCs isolated from FMDV-infected swine did not show down-regulation of MHC class II molecules. The fact that we have not detected any viral replication in MoDCs *ex vivo*, may indicate that down-regulation of MHC class II molecules requires virus replication or at least certain level of viral replication. However, we cannot discard that FMDV replicates in MoDCs *in vivo* but under the limit of our detection method or using a more sensitive method to detect virus. This observation indicates that FMDV infection interferes either the transcription or translation of MHC class II molecules. The resultant lack of MHC class II molecules ultimately contributes to the inability of MoDCs to function properly. It is worth noting that interference with MHC class II surface expression has also been reported for HIV, cytomegalovirus and LCMV [40], [44], [45].

The failure of FMDV-infected MoDCs *in vitro* and MoDCs *ex vivo* from FMDV-infected swine to stimulate T cell proliferation is associated with the production of IL-10 by MoDCs. MoDCs exhibit the most potent substantial increase in IL-10 production during persistent infections and may contribute to immunosupression. Increased IL-10 production by APCs is also observed during HIV and hepatitis virus C infections and has been shown to specifically down-regulate T-cell responses [46], [47], [48], [49], [50]. Because IL-10 can induce T-cell unresponsiveness when present during T-cell activation [51], the ongoing interaction of immunosuppressive IL-10 producing MoDCs with T cells probably leads to the loss of T-cell responsiveness. A very clear example is the persistent infection of mice with LCMV, characterized by an inactivation of antiviral T cells, which show a significant up-regulation of IL-10. *In vivo* blockade of IL-10 receptor resulted in rapid resolution of the persistent infection [17], [18]. Thus, our data show that FMDV infection induces IL-10 production by MoDCs *in vitro* and *in vivo*, and therefore inhibits T-cell dependent responses. One of the most noteworthy findings of this study is that *in vitro* antibody blockade of IL-10 completely or partially restored T cell activation, implying an important role of IL-10 in T cell suppression. These data are consistent with previous reports in a mouse model system that demonstrated that FMDV infection in mice induces production of IL-10 *in vivo* [36], [52]. Although the *in vivo* role of IL-10 is generally immunosuppressive, this cytokine plays an important stimulatory role in the function of B-lymphocytes and the production of antibodies by B1 lymphocytes during the development of an immune response against antigens from pathogens. In agreement to that, some researchers have reported the IL-10 B-cell stimulatory role in mice immunized with glutamate dehydrogenase from *Trypanosoma cruzi* [53].

In conclusion, we have shown for the first time a role of IL-10 in pathogenesis of FMDV in the natural host. During acute infection of swine with FMDV, a viremic phase occurs followed by a rapid neutralizing antibody response that eliminates the infection. In acute infections, a strong virus-specific CD4^+^ T cell activation may result in heightened early polyclonal B cell activation, which competed with virus-specific B cell activation and the formation of neutralizing antibodies. A reduction in CD4^+^ T cell function and/or in CD4^+^ T cell numbers decreases polyclonal B cell stimulation, apparently by concentration of specific T cell help onto virus-specific B cells [54], stimulating the production of neutralizing antibodies. Such a scenario may be the case for FMDV infection. Based on our data, we propose that IL-10 produced upon FMDV infection suppresses CD4^+^ T cell activity, as we have previously shown [29] during acute infection, promoting the activation of virus-specific B cells to produce neutralizing antibodies, clearing the viral infection.

## Materials and Methods

### Animals and virus

Twenty Large White×Landrace female pigs of 8–9 weeks old were used in this study. The animals were housed in isolation at the Centro de Investigación en Sanidad Animal (CISA-INIA) in Spain. Sixteen animals were inoculated by intradermal route in the coronary band of the right front limb with 10^5^ PFU of FMDV C-S8c1 in 0.5 ml of PBS. The FMDV C-S8c1 is a plaque-purified derivative of the natural isolate C_1_-Sta Pau-Spain 70, a representative of the European subtype C_1_ FMDV [55]. The preparation of gradient-purified binary-ethylenimine (BEI)-inactivated FMDV C-S8c1 has been done as previously described {Bahnemann, 1975 #2026}. The animals were euthanized in batches of two animals at 1, 2, 3, 5, 7, 10, 14 and 17 days post-inoculation (dpi). Four pigs were used as uninfected controls, housed in different boxes, and killed at the end of the experiment: 2 were non-inoculated controls (NI) and 2 received an injection of 0.5 ml sterile PBS in the coronary band of the right front limb. All experiments involving animals were approved by the ethical review committee at the Centro de Investigación en Sanidad Animal (CISA-INIA), following guidelines set forth the European Union (Directive 86/609/EEC).

### Generation of porcine monocyte-derived DCs

Peripheral blood mononuclear cells (PBMCs) were prepared from whole blood of FMDV C-S8c1 infected and uninfected control pigs as previously described [29]. Briefly, PBMCs were obtained from heparanized blood of specific pigs by density gradient centrifugation at 1,000× g for 25 min, over Ficoll-Plaque (GE Healthcare, Madrid, Spain). Cells expressing CD172^+^ were purified from total PBMCs (with >97% purity) using an anti-CD172 porcine pan-myeloid cell marker (monoclonal antibody BL1H7, provided by J. Dominguez, INIA, Madrid, Spain) by magnetic cell sorting using a MACS system (Miltenyi Biotec, Bergisch Gladbach, Germany) and positive selection LS column. These cells were cultured for 6 days in RPMI medium supplemented with 10% (v/v) fetal calf serum and the recombinants porcine cytokines granulocyte-macrophage colony-stimulating factor (rpGM-CSF) at 50 ng/ml (Invitrogen, Carlsbad, CA, USA) and recombinant porcine interleukin-4 (rpIL-4) (50 ng/ml, Biosource, Nivelles,Belgium) to allow differentiation of DCs. Fresh cytokines were added every 2 days. To drive DC to complete maturation, non-adherent cells were collected at day 6 of incubation and resuspended in culture medium containing rpGM-CSF, rpIL-4 and Tumor Necrosis Factor-alpha (TNF-α) (5 ng/ml, Sigma, St Louis, MO, USA), and incubated for another 24 hours.

### Cell infection

Total PBMCs were infected before CD172^+^ cells selection. CD172^+^ cells were either infected after magnetic cell separation (D0) or after 5 days of culture in rpGM-CSF and rpIL-4 (D5). In either case, cells were infected in six-well plates at an MOI of 10 PFU/cell. Viral attachment was performed during one hour at 37°C, and subsequent washing with phosphate buffer pH 6.0 to eliminate any virus bound to the cellular membrane that could account for the infectivity. Supernatants of FMDV infected DC were titrated by plating serial dilutions on BHK-21 cells monolayers. After 1 h adsorption at 37°C in 5% CO_2_, the cells were overlaid with DMEM containing 2% fetal calf serum, 0.5% agar and DEAE-dextran (0.045 mg/ml) [56]. Plaques were visualized 24 h post-infection (hpi) by crystal violet staining of formaldehyde (7% vol/vol) fixed cells. For infection of confluent BHK-21 cell monolayers with supernatants of FMDV-infected DCs in liquid medium, the cell culture medium was removed and the supernatant added onto the cell monolayer. Virus was adsorbed to cells for 1 h at 37°C in 5% CO_2_ with gentle rocking every 15 min; then the cells were overlaid with DMEM containing 2% fetal calf serum. Infections were allowed to proceed until cytopathology was complete, or in case of lack of cytopathology, the supernatant of this infection was used to infect fresh BHK-21 monolayers.

### Viral RNA quantification

RNA was extracted from FMDV infected DCs by treatment with Trizol (Invitrogen) according to the instructions of the manufacturer [57]. FMDV RNA quantification was performed by real time RT-PCR using the LightCycler instrument (Roche, Indianapolis, IN, USA) and the RNA Master SYBR green I kit (Roche), as specified by the manufacturer. Quantification was relative to a standard curve obtained with known amounts of FMDV C-S8c1 RNA, using a procedure that has been described previously described [58], [59].

### Cytokine ELISA

Cytokine concentrations were determined in cell culture supernatants and serum from infected animals. IL-10, IFN-γ and IFN-α was determined by sandwich enzyme-linked immunosorbent assay (ELISA) according to the manufacturer's directions (Biosource). It was developed with 3, 3′, 5, 5′, tetramethylbenzidine (TMB) from Sigma. The absorbance at 450 nm was measured in an ELISA reader (VersaMax, Molecular Devices, Sunnyvale, CA, USA). Cytokine concentrations were calculated based on the optical densities obtained with the standards.

### Flow cytometric analysis

Cells were pelleted by centrifugation and resuspended in staining buffer (PBS containing 2% [vol/vol] fetal calf serum and 0.2% [wt/vol] NaN_3_) for flow cytometry. To analyze the expression of cell surface molecules we used monospecific antibodies, fluorochrome dyes and flow cytometry. The primary antibodies used were mouse-anti SLA-IIDR-biotin (clone 1F12, provided by J. Domínguez, INIA, Madrid, Spain) and human CD152 (CTLA-4) murine immunoglobulin/Biotin fusion protein (IG2a), that binds swine CD80/86, purchased from Ancell. In a second step, streptavidin conjugated to APC or PE (BD Pharmingen) was used. After staining, cells were fixed in PBS/1% fetal bovine serum/4% PFA (wt/vol). Cells were acquired using a FACSCalibur flow cytometer (Becton Dickinson, Franklin Lakes, NJ, USA). Dead cells were excluded on the basis of forward and side light scatter. Data were analysed with CellQuest software (Becton Dickinson).

### Determination of cell viability and apoptosis

Viability and apoptosis in MoDCs cultures were determined using the Annexin V-PE apoptosis detection kit from Pharmingen (BD Pharmingen) according to the manufacturer's instructions. Briefly, 1×10^5^ cells were centrifugated and resuspend in 100 µl of binding buffer in the presence of 5 µl of Annexin-V-PE and 5 µl of 7-AAD. After 15 min incubation at room temperature, 400 ml of binding buffer was added, and cells were analyzed by flow cytometry using a FACSCalibur flow cytometer (Becton Dickinson, Franklin Lakes, NJ, USA).

### Measurement of T cell stimulation

The ability of DCs to act as accessory cells for T cell stimulation in a one-way mix lymphocyte reaction (MLR) was compared in a [^3^H]thymidine incorporation assay as described [29]. Briefly, the responding cells were CD3^+^ T cells purified by magnetic sorting from total PBMCs using MACS system (Miltenyi Biotec) and mouse-anti swine-CD3 monoclonal antibody (provided by J. Domínguez, INIA, Madrid, Spain). Accessory cells were DCs uninfected or infected with FMDV at D0 or D5. Also, DCs isolated from FMDV infected swine were used. DCs were placed in suspension and γ-irradiated (2,000 rads) after which 2×10^3^ cells were mixed with different number of responder cells in a total volume of 200 µl. The cells were then incubated at 37°C for 3 days. During the final 18 hours of culture, cells were pulsed with 1 µCi of [^3^H]-thymidine added to each well and then harvested. The amount of radioactivity incorporated was determined by using a liquid scintillation beta counter (Beckman Instruments, Inc.Madrid, Spain). Results shown are the mean [^3^H]-thymidine incorporation of triplicate wells.

### Blocking of induced IL-10

Co-cultures of CD3^+^ T cells and DCs were cultured for three days in the presence of neutralizing IL-10 antibody, used at a final concentration of 3 µg/ml (clone 148801, R & D Systems, Minneapolis, MN, USA). The co-cultures were incubated with an irrelevant antibody (mouse anti-porcine IgG2) as a negative control. During the last 18 hours of culture, cells were pulsed with 1 µCi of [^3^H]-thymidine added to each well and then harvested. The amount of radioactivity incorporated was determined by using a liquid scintillation beta counter (Beckman Instruments, Inc.). Results shown are the mean [^3^H]-thymidine incorporation of triplicate wells.

### Immunohistochemistry and confocal microscopy

For immunohistochemical studies, cells were added to poly-lysine coated chamber slides (Lab-Tek) and fixed with 4% paraformaldehide (PFA) for 10 min., permeabilized with 0.1% Triton X-100 in PBS for 10 min and blocked with avidin/biotin blocking solution (Vectorlabs, Burlingame, CA, USA). Mouse anti-FMDV 3D (MAb 3H11, gently donated by E. Brocchi, IZS Brescia, Italy) (1:100) was added and incubated at room temperature for 1 hour. As secondary antibodies, anti-mouse IgG conjugated to Alexa-594 (Invitrogen) was used at a 1∶100 dilution. Stained cells were visualized using a LSM 510 META confocal microscope (Zeiss) and 63× oil objective. DAPI staining was used to visualize nuclei.

### Statistical analyses

Data handling, analyses and graphic representation was performed using Prism 2.01 (GraphPad Software Inc. SanDiego, CA, USA). Statistic differences were determined using a Student *t* test or a one-way ANOVA (p<0.05).
